# A chatbot-based intervention with ELME to improve stress and health-related parameters in a stressed sample: Study protocol of a randomised controlled trial

**DOI:** 10.3389/fdgth.2023.1046202

**Published:** 2023-03-01

**Authors:** C. Schillings, D. Meissner, B. Erb, D. Schultchen, E. Bendig, O. Pollatos

**Affiliations:** ^1^ Department of Clinical and Health Psychology, Ulm University, Ulm, Germany; ^2^ Institute of Distributed Systems, Ulm University, Ulm, Germany; ^3^ Department of Clinical Psychology and Psychotherapy, Ulm University, Ulm, Germany

**Keywords:** chatbot, intervention, stress, interoception, mindfulness, digital health

## Abstract

**Background:**

Stress levels in the general population had already been increasing in recent years, and have subsequently been exacerbated by the global pandemic. One approach for innovative online-based interventions are “chatbots” – computer programs that can simulate a text-based interaction with human users *via* a conversational interface. Research on the efficacy of chatbot-based interventions in the context of mental health is sparse. The present study is designed to investigate the effects of a three-week chatbot-based intervention with the chatbot ELME, aiming to reduce stress and to improve various health-related parameters in a stressed sample.

**Methods:**

In this multicenter, two-armed randomised controlled trial with a parallel design, a three-week chatbot-based intervention group including two daily interactive intervention sessions *via* smartphone (á 10–20 min.) is compared to a treatment-as-usual control group. A total of 130 adult participants with a medium to high stress levels will be recruited in Germany. Assessments will take place pre-intervention, post-intervention (after three weeks), and follow-up (after six weeks). The primary outcome is perceived stress. Secondary outcomes include self-reported interoceptive accuracy, mindfulness, anxiety, depression, personality, emotion regulation, psychological well-being, stress mindset, intervention credibility and expectancies, affinity for technology, and attitudes towards artificial intelligence. During the intervention, participants undergo ecological momentary assessments. Furthermore, satisfaction with the intervention, the usability of the chatbot, potential negative effects of the intervention, adherence, potential dropout reasons, and open feedback questions regarding the chatbot are assessed post-intervention.

**Discussion:**

To the best of our knowledge, this is the first chatbot-based intervention addressing interoception, as well as in the context with the target variables stress and mindfulness. The design of the present study and the usability of the chatbot were successfully tested in a previous feasibility study. To counteract a low adherence of the chatbot-based intervention, a high guidance by the chatbot, short sessions, individual and flexible time points of the intervention units and the ecological momentary assessments, reminder messages, and the opportunity to postpone single units were implemented.

**Trial registration:**

The trial is registered at the WHO International Clinical Trials Registry Platform *via* the German Clinical Trials Register (DRKS00027560; date of registration: 06 January 2022). This is protocol version No. 1. In case of important protocol modifications, trial registration will be updated.

## 1. Background

Stress levels in the general population had already been increasing in recent years, and have subsequently been exacerbated by the global pandemic. In particular, 64% of a representative German adult sample feel stressed at times and 26% feel stressed frequently (1). 77% of the latter stated that they experience life today as more stressful than 15–20 years ago. As the two central reasons for the high stress experience, school, studies, or work as well as high demands on themselves were reported. These are followed by the illness of a closely related person as a source of stress which might be associated with the COVID-19 pandemic (1). Systematic reviews and meta-analyses based on studies investigating stress, anxiety, and depression prevalence among the general population during the COVID-19 pandemic worldwide (2, 3), showed a mean prevalence of stress between 30% and 37%, 24 to 31% for the prevalence of anxiety, and 28% to 34% for the prevalence of depression. Consistently, the numbers of mental disorders increase, resulting in the main reason for days absent due to illness (4). Moreover, the International Classification of Diseases, 11th Revision (ICD-11), was extended by a category of stress-related mental disorders (5), which underlines the relevance of stress and the need for interventions of stress reduction.

One approach to cope with stress and different related psychological disorders is mindfulness-based interventions. Mindfulness has been conceptualized as a state of being aware and focused on the present moment in an open, accepting and non-judgmental way (6–9). Previous meta-analyses and reviews showed positive effects of online mindfulness-based interventions on mental health outcomes such as decreases in perceived stress (e.g., 10–12), anxiety (e.g., 11, 12), depression (e.g., 11, 12) as well as increases in mindfulness (e.g., 10, 11) and well-being (e.g., 11).

Another health-related, closely related construct to stress is interoception. Interoception is defined as the process of the nervous system of sensing, interpreting, and integrating internal bodily signals (13) such as cardiovascular, respiratory, or gastrointestinal signals to a moment-by-moment internal bodily landscape across conscious and unconscious levels (14, 15). According to a recent classification model of interoception by 16 (16), a distinction between accuracy and attention to interoceptive signals (i.e., factor 1) and between objective measures and self-reported beliefs concerning interoceptive signals (i.e., factor 2) is made. Previous research indicated associations between interoceptive abilities and stress (e.g., 17–21). In particular, a study by Schultchen and colleagues (20) found that a decreased objective interoceptive accuracy is associated with higher long-term stress. Moreover, several studies showed that interoceptive abilities are associated with emotional abilities (e.g., 22, 23) and that they are impaired in diverse mental disorders such as anorexia nervosa (e.g., 24), depression (e.g., 25), or schizophrenia (26). An increasing body of research indicates that interoceptive abilities can be trained, for example, *via* mindfulness-based interventions (e.g., 27, 28), body-focused training such as power posing (29), or heartbeat perception training (30, 31). So far, online interventions to improve interoceptive abilities are sparse. An ongoing study (32) investigates the effects of a guided online mindfulness-based intervention on stress, interoceptive abilities and further health-related parameters. Integrating such trainings into everyday life might be a promising approach (31).

In the past few years, there has been a growing interest in the use of and the research about chatbots (33–35). A “chatbot” or conversational agent is defined as a computer program, which is able to simulate a text-based interaction with human users *via* a conversational interface (e. g., *via* website or smartphone; 36, 33). Chatbots can be classified into different categories such as application domain (i.e., domain-specific knowledge support), service provided (i.e., scripted dialogs vs. query interface for information retrieval), or according to the response generation method (i.e., rule-based selection of pre-defined text components vs. natural language processing and machine learning-based responses; 33, 37). Other characteristics of chatbots comprise the type of interaction interface, i.e., the device on which the chatbot interacts with the user (e.g., mobile application, web browser), or the input and output modality (written, spoken, or mixed), among others (38).

Chatbot-based interventions provide advantages such as low-threshold and anonymous use, flexibility regarding time and location of use, and cost-effectiveness; therefore, they could be integrated easily into everyday life (39–43). Moreover, especially the effects due to guided online interventions (i.e., intervention contents are accompanied or provided by a guide such as an e-coach or even a chatbot) to improve mental health need to be highlighted (44, 11), as they showed higher adherence rates (39, 45, 46) and were more effective in terms of symptom severity reduction (47) as compared to unguided interventions. Social motivation might be an essential factor in the effectiveness of chatbot-based interventions (34, 48). Mental health apps including psychoeducation, also *via* a chatbot, have shown to provide the potential to decrease stigma, e.g., *via* the use of the chatbot and educational strategies (49), and to increase mental health literacy (39, 42, 50, 51). Particularly, lacking awareness of available support was revealed as a substantial barrier to mental health access in young people (52, 53). Additionally, previous research on chatbots supports their potential to deliver psychoeducation and to promote self-adherence (54).

In the application of chatbots to improve mental health, research on the efficacy of chatbot-based interventions is still sparse (37, 38, 55). A mixed-method systematic review based on chatbot-based interventions for mental health (56) showed significant decreases in psychological distress with effects ranging from small (*d* = 0.24) to very large (*d* = 2.0). In particular, the improved outcomes comprised depression (57–59), psychological distress (60), anxiety (58), fear of heights (61), and positive affect (58, 60). Moreover, these findings are also summarized in the reviews by (54) and (55), inter alia, including the findings of increased well-being (60). In contrast, for example, a pilot study by (62) found neither significant improvements in perceived stress nor in psychological well-being in a non-clinical sample due to a two-week smartphone-based intervention. This intervention was based on positive psychology and cognitive-behavioural therapy provided by a chatbot in comparison to a wait list control group. Nevertheless, this sample included only 28 participants, and, results revealed significant effects when only including the adherent participants (i.e., in this study, those participants with at least 25% activity and not being inactive for more than 7 days or more in a row). Similarly, the findings by (63) showed significant reductions in self-reported symptoms of depression in high users compared to the low user group. In contrast, for a two-day chatbot-based therapeutic writing intervention aiming to improve psychological well-being, good feasibility, but no effects on well-being were demonstrated (64, 65). Both improved well-being and stress reduction due to a three-week chatbot-based stress management intervention were reported in a sample of young adults (66). Comparably, a recent study (67) found significantly reduced stress levels and decreased anxiety in students due to a 4-week chatbot-based intervention based on cognitive behavioral therapy, mindfulness techniques, and positive psychology. It needs to be considered that both studies (66, 67) were uncontrolled. To sum up, previous studies differ in various aspects such as the study design, the intervention duration, outcome assessments, primary goals of the chatbot, type of communication technology, input and output modality, and their samples. There is still a lack of standard measures and randomized controlled trials of chatbot-based interventions in the mental health area (55, 68, 38, 35). Moreover, standards for chatbot-based mental health apps are missing (39, 42). Consequently, more structured randomized controlled trials on chatbot-based interventions to improve specific parameters of mental health such as stress or anxiety based on standard measures and guideline-based chatbots are needed.

We developed a three-week chatbot-based intervention *via* smartphone comprising two daily short sessions (á 10–20 min.) for a sample with medium to high stress levels, aiming to improve stress and health-related parameters such as interoception and mindfulness. The chatbot is named ELME, a gender-neutral name as an acronym for Everyday-life Mindfulness Experience. The intervention duration was determined based on previous studies investigating chatbot-based interventions ranging from two to four weeks (e.g., 67, 69, 62; 60, 66) and reported preferences of an adult sample for short online sessions (70). Considering the target group of the intervention, namely, a stressed sample, short sessions were found to be effective to reduce perceived stress and individuals seem to use intervention exercises more frequently if they take less time (71).

According to the CONSORT Consolidated Standards of Reporting Trials (CONSORT) 2010 Guidelines for randomized controlled trials (72, 73) and the according extension for randomised pilot and feasibility trials (74), we conducted several pilot phases and a feasibility study (DRKS00025446) based on a sample size of *n* = 44 with usability as assessed *via* the mHealth App Usability Questionnaire (75) as the primary outcome variable and the design of the actual study. Results showed that the chatbot-based intervention is a feasible and flexible tool. User feedback was implemented to an optimised version of the chatbot used in the current study. Aiming to increase adherence, this adapted version of ELME includes even shortened units, the setting to switch the training time slots for the next day and an adaptation of the favoured typing speed of ELME. The present study investigates the effects on diverse health-related and user-oriented parameters.

We hypothesize that:

(1) the primary outcome perceived stress will be reduced in the intervention group compared to the treatment as usual control group.
(2) the secondary outcomes interoception, mindfulness, and psychological well-being will be improved.

Furthermore, we examine potential changes in secondary outcomes as health-related and related to the primary and main secondary outcomes, but not directly in the intervention targeted variables, e.g., depression, anxiety, emotion regulation, stress mindset and test for potential modifying effects on an exploratory level. For example, stress mindset could be a moderating factor of perceived stress (76, 77). Moreover, similar health-related measures had been investigated in the reported previous online or chatbot-based intervention studies (e.g., 55, 65–67). Additionally, based on previous research, we investigate user-oriented parameters such as usability, satisfaction with the intervention, and, lastly, adherence and potential dropout reasons, to potentially further improve the intervention for future research.

## 2. Methods and analysis

### 2.1. Study design

The present study is a two-arm, parallel randomized controlled trial with an intervention group compared to a control group receiving treatment as usual. The intervention group receives a three-week online-based intervention guided by the chatbot ELME. The control group receives no content and just answers the questionnaires and the ecological momentary assessments. Treatment as usual for the control group was chosen based on methodological recommendations for randomized controlled trials and psychological interventions (71, 78, 79). Primary and secondary outcomes will be assessed in both groups at screening (t0), pre-intervention (t1), daily during the intervention (between t1 and t2), post-intervention (t2), as well as follow-up three weeks after t2 (t3). The study will be conducted in accordance with the Consolidated Standards of Reporting Trials (CONSORT) 2010 Guidelines for Randomized Controlled Trials (71, 73). The study protocol corresponds the recommendations of the “Standard Protocol Items: Recommendations for Interventional Trials” checklist for clinical trial protocols (SPIRIT; 80).

### 2.2. Eligibility criteria

Participants will be included in the present study if they (a) are 18 years or older, (b) have sufficient knowledge of the German language, (c) own a smartphone (Android or iOS) with internet access, (d) possess a valid German phone number, (e) possess a valid mail address, (f) experience middle to high perceived stress (PSS-10 score ≥ 14, assessed at t0), (g) are not diagnosed with any mental disorder, (h) do not currently undertake psychotherapy, and (i) do not currently participate in another mental health online-intervention.

### 2.3. Setting and recruitment

Recruitment of the study has started in February 2022 and will be continued until the targeted sample size of *N *= 130 has been reached. Recruitment takes place online and offline targeting German speaking people. Offline recruitment strategies comprise flyers and posters at different public places such as universities, fitness centers, educational institutions, corporate health management, psychosocial counselling services, and city libraries in Germany. Online recruitment will be implemented *via* e-mail distribution lists, e.g., in the area of occupational health management, universities, postings on social media (e.g., LinkedIn), online self-help groups on social media. Apart from the possibility to take part in a chatbot-based intervention for free, participants receive the chance to win a € 25 gift card from an online shop or, as a student participant, to receive 5 course credits as expense allowance for completing the questionnaires. Furthermore, both intervention and control participants receive the option to get access to two relaxing exercises and to get individual summaries regarding the change in their health-related parameters in the course of t1 to t3 after completing the t3 questionnaire.

### 2.4. Study procedure

**Figure 1:**
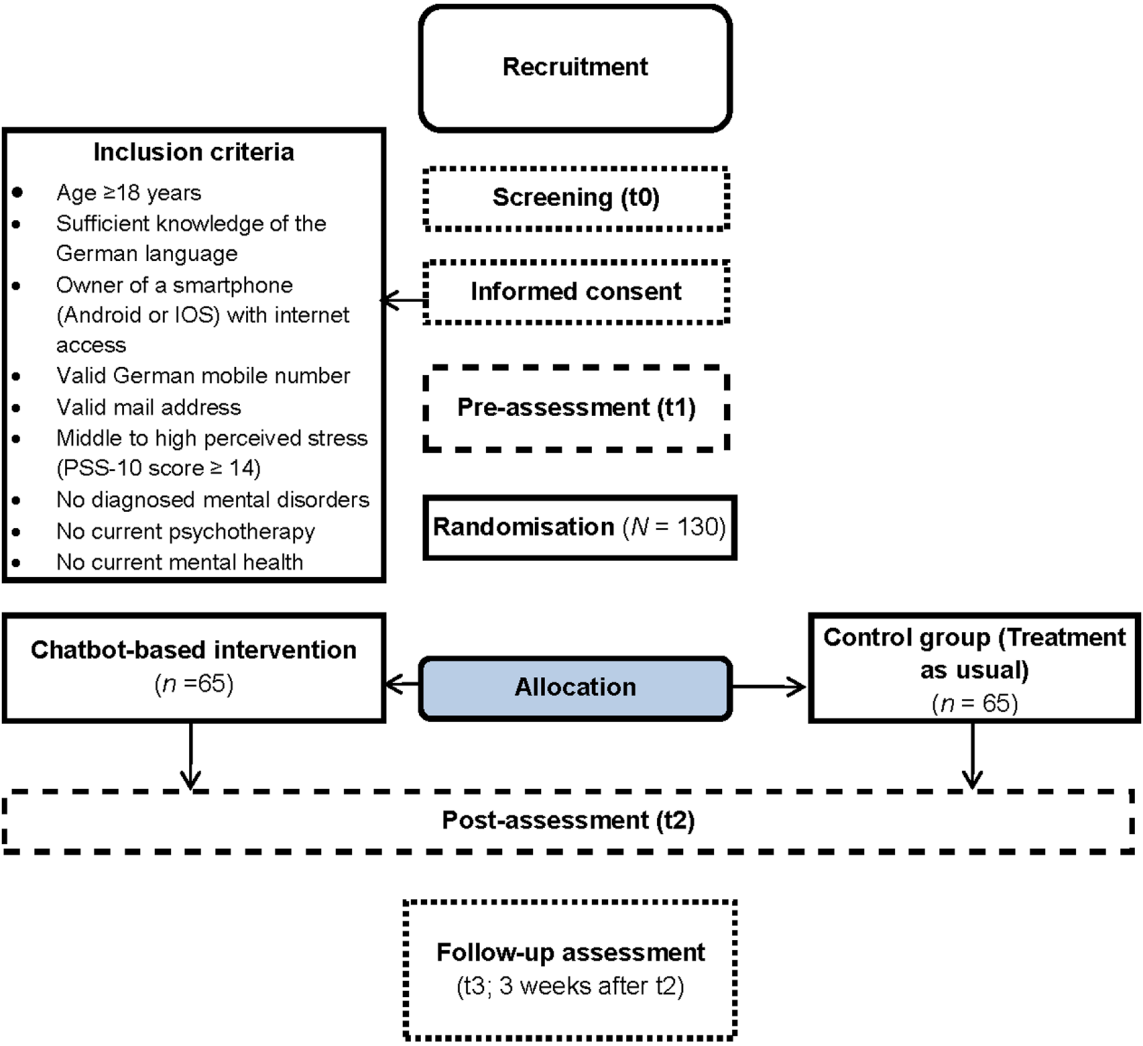
Flow chart of the planned study procedure.

**Figure 2:**
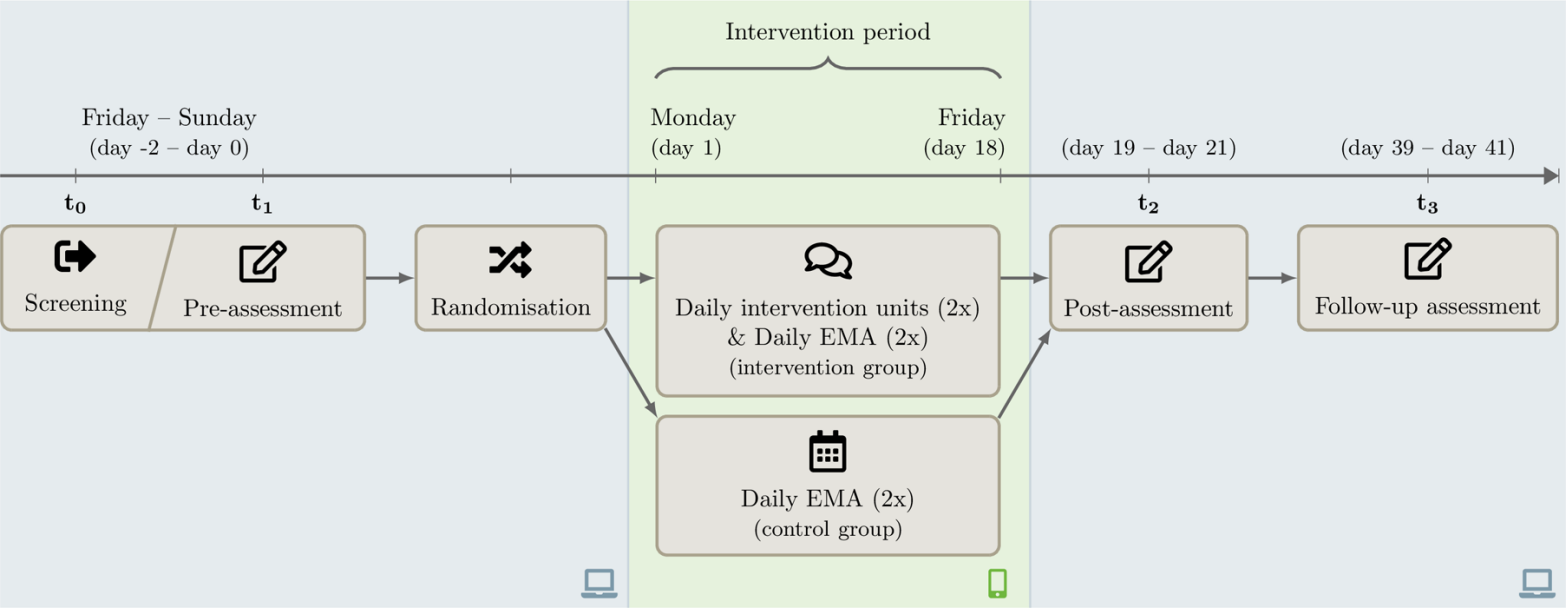
Participant timeline for the intervention and the control group. *EMA,* Ecological momentary assessment.

Interested individuals can access to the screening questionnaire (t0) *via* the landing page, after they have registered with their mail address and their phone number and verified those. The landing page can be reached *via* a link or a QR code. If participants fulfill the inclusion criteria (see Figure 1), they will be forwarded to the pre-intervention questionnaire (t1). As the first part of the t1 questionnaire, participants obtain and have to agree with the informed consent of the study. The questionnaires t0-t3 take place *via* the online survey tool Unipark (https://www.unipark.com). After completing the t1 questionnaire, included participants will automatically receive an SMS with a personalized link to the specifically developed chatbot platform. By linking the participants' sessions to their mobile numbers, the phone is used as the sole authentication factor and no other credentials are required for personalized access. Apart from offline recruitment strategies, the study will be fully conducted online. Excluded participants are informed that they could not take part in the study and receive alternative contact institutions such as psychosocial contact institutions or platforms to find psychotherapists. Ecological momentary assessments take place *via* the chatbot platform. Figure 1 represents the planned study procedure. A participant timeline for the intervention and the control group is depicted in Figure 2.

### 2.5. Randomisation and blinding

After completing the t1 questionnaire, the platform automatically assigns participants randomly to the intervention group or to the control group. The assignment is based on a platform-internal pseudorandom number generator with an allocation ratio of 1:1. Data analyses are performed with pseudonymous data, where the analysts do not learn about the participants' identities. Primary and secondary outcome analysis are performed with a blinded data set, which conceals the group allocation while keeping all participants of the same group in the same blinded group.

### 2.6. Intervention

ELME is a rule-based, conversational chatbot and was developed by members of the Department of Clinical and Health Psychology and the Institute of Distributed Systems at Ulm University. This type of technology is a self-developed, web-based chatbot platform, provided *via* smartphone with SMS notifications. The input modality is written; the output modality is also written, except audio files provided by the chatbot.

The chatbot-based intervention aims at stress reduction and improving health-related parameters such as interoception and mindfulness. In particular, the intervention contents mainly address the constructs of stress, interoception and mindfulness as well as their association, provided *via* psychoeducation and exercises (e.g., audio files such as breathing exercises) in a real-time dialogue with the chatbot. The intervention units are offered twice a day with one session in the morning and one session in the afternoon or evening over the course of three weeks, depending on the self-selected time of a participant. The times of the units could be switched daily *via* a settings menu in the chatbot platform. In the weekends, only one session takes place, the day can be freely selected by each participant. Importantly, the contents and exercises aim to be closely related to everyday life. Therefore, the chatbot asks questions related to current situations of the participants and exercises are designed short (10–20 min). The gender-neutral persona of the chatbot is characterized by a friendly demeanor as an empathetic companion with expertise in mental health. ELME's communication style can be described as calm, tolerant, supportive, and appreciative. For each start of a session with ELME, the participant receives an SMS. Furthermore, participants receive reminder SMS to fulfill the units with ELME and they could postpone single exercises to one hour later. Participants have to complete each session within three hours; otherwise ELME aborts the session automatically to assure the course of upcoming sessions. Moreover, the chatbot platform menu provides answers to frequently asked questions, a download function for the audio files and summaries of the single exercises.

**Figure 3:**
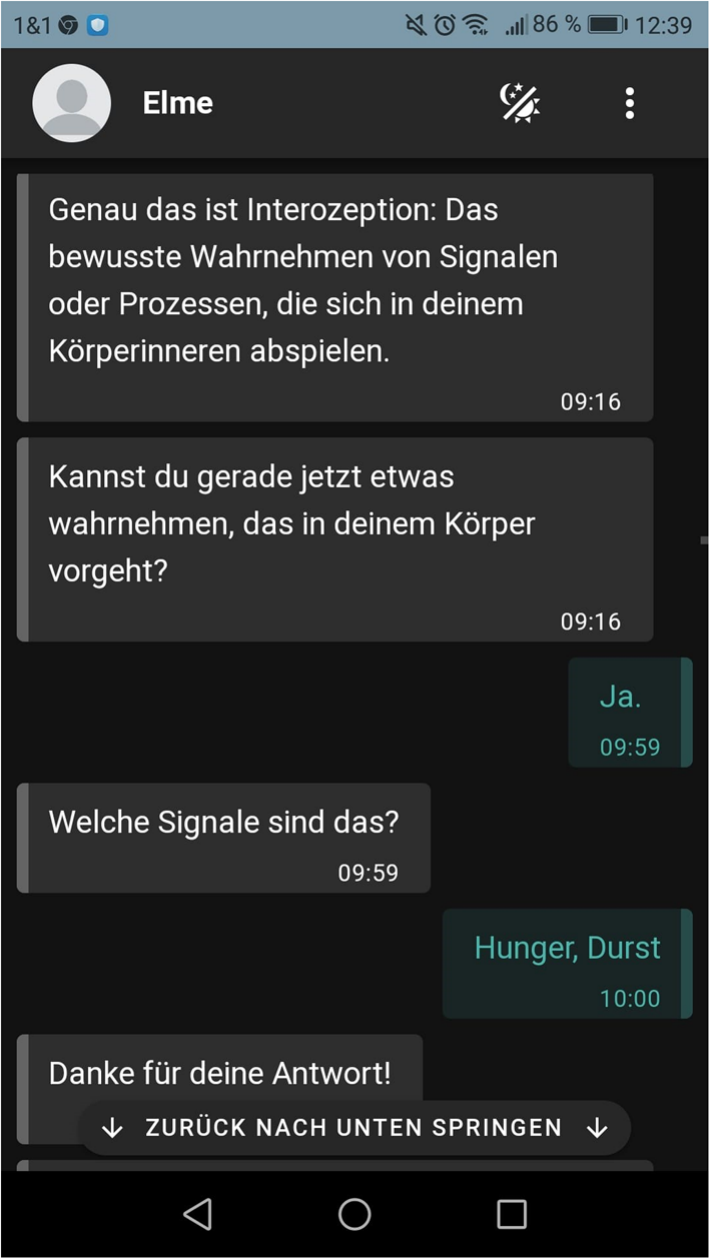
Sample dialogue of the chatbot interacting with a participant.

The contents are based on approaches of mindfulness-based stress reduction (81–83), stress management (84, 85), Acceptance and Commitment Therapy (86, 87), heartbeat perception exercises derived from the heartbeat tracking task (88), and psychoeducative elements of a guided online mindfulness-based intervention called “StudiCare Mindfulness” (32). An overview of the alternating intervention contents representing the modules “stress”, “interoception”, “mindfulness” or the “association of stress, interoception, and mindfulness” is shown in Table 1. In every training session, ELME introduces the participants to a main topic by psychoeducation, provides everyday examples and corresponding exercises. Aiming to make the participants integrate the topics into their everyday life, central intervention contents and exercises are repeated. They are engaged in the interaction with ELME by answering questions with pre-defined response alternatives, on a numeric slider or *via* an open text. Based on a fixed set of rules, the communication logic is implemented as a finite-state machine. On each incoming message sent by the participant, ELME responds with an appropriate answer. ELME purposefully and frequently involves users in the conversation to ensure their active participation, also reminding of responding by diverse text phrases. A sample dialogue of the chatbot interacting with a participant is depicted in Figure 3 (in German language).

**Table 1:** Overview of the intervention contents.

Module	Aims and content	Presentation form
Stress		
Introduction into the construct stress	Psychoeducation; understanding the benefit of the intervention	Chat dialogue
Stress Traffic Light	Instruction of the Stress Traffic Light (84), training of stress perception and management	Chat dialogue
Stress reliever	Psychoeducation and reflection of stress relieving methods	Chat dialogue
Theory unit regarding stress	Impulses targeting stress management, coping strategies	Chat dialogue
Interoception		
Introduction into the construct interoception	Psychoeducation (e.g., 15, 89) understanding the benefit of the intervention	Chat dialogue
Heartbeat tracking task in sitting and lying position; walking	Exercise targeting interoception / heartbeat perception; adaption to the heartbeat tracking task (88, 27, 90)	Chat dialogue, audio file
Mindfulness		
Introduction into the construct mindfulness	Psychoeducation (e.g., 8) understanding the benefit of the intervention	Chat dialogue
Query of the most (non-)mindful moment of the day	Mindfulness exercise in every-day life, reflection	Chat dialogue
Body Scan	Mindfulness exercise	Audio file
Mindful drinking of water	Mindfulness exercise/Perception of satiety/pleasure	Chat dialogue
Mindful walk	Mindfulness and relaxation exercise	Chat dialogue
Association of stress, interoception & mindfulness		
Breathing exercise	Stress reduction and mindfulness exercise	Audio file
Query of satiety	Exercise targeting interoception/mindful perception of satiety	Chat dialogue
Association of stress, interoception and mindfulness	Psychoeducation, e.g. by explaining empirical findings (20, 18, 17, 91)	Chat dialogue
Free sports activity	Health promotion by physical activity as a positive impact on interoception, mindfulness and stress reduction (91)	Chat dialogue
Final summary	Reflection of the whole intervention, repetition	Chat dialogue, PDF file for downloading
Bouquet of flowers	Summary and explanation of all intervention exercises	PDF file

### 2.7. Treatment as usual control group

The control group receives no treatment. Participants of the control group only do the assessments, i.e., the questionnaires t0–t3 and daily ecological momentary assessments.

### 2.8. Outcome assessment

#### 2.8.1. Primary outcome: stress

To screen the participants concerning their acute perceived stress levels as an inclusion criterion, the 10-item version Perceived Stress Scale (PSS-10; 92) is used. Acute perceived stress regarding the last month is rated as the degree to which situations in one's life are rated as stressful on a scale ranging from 0 = “never” to 4 = “very often”. Within the questionnaires from t1 to t3, the 4-item short scale (PSS-4) is implemented. The ratings on both scales are quantified as sum scores with higher scores representing higher perceived stress. The German version of the PSS-10 demonstrated good internal consistency (Cronbach's Alpha = .84; 93) and the PSS-4 exhibited acceptable and reliable psychometric properties across cultures (Cronbach's Alpha = 0.77, 94).

#### 2.8.2. Secondary outcomes

##### 2.8.2.1. Self-reported interoceptive accuracy

Interoception, specifically, self-reported interoceptive accuracy, will be assessed *via* German versions of the Interoceptive Accuracy Scale (IAS; 95) and the subscale “Awareness” of the Body Perception Questionnaire (BPQ; 96). A self-translated German version of the IAS is used. The IAS consists of 21 items asking the participants how well they believe they can perceive specific bodily sensations on a 5-point Likert scale ranging from 1 = “disagree strongly” to 5 = “strongly agree”. Based on calculated sum scores (range: 21–105), higher scores reflect greater self-reported interoceptive accuracy. The internal consistency of the IAS is high with Cronbach's Alpha of 0.88 (95, 97). The BPQ comprises 45 items describing body processes which should be rated concerning the awareness during most situations on a 5-point Likert scale ranging from 1 = “never” to 5 = “always”. A mean score of the subscale “Awareness” is calculated. Based on a mean of 50 and a standard deviation of 10, t scores represent standardized values according to a normal distribution. High internal consistency with Cronbach's Alpha of 0.92 (98) and 0.97 (97) was shown.

##### 2.8.2.2. Ecological momentary assessment

Ecological momentary assessment includes questions on acute perceived stress, body awareness, interoception, and mood in the according moment. Participants have to answer the questions on a visual analogue scale based on horizontal rating sliders ranging from 0 “ = not at all” to 100 = “very much”. Comparably to the ecological momentary assessment in a previous study (99), acute perceived stress is assessed *via* two adapted items for the momentary use of the Perceived Stress Scale Short form (PSS-4; 92): “Do you feel that things are going your way?” and “Do you find you can cope with all the things that you have to do?”. Furthermore, two items addressed the body awareness: “How present do you feel at the moment?” and “How aware are you of your own body at the moment?” (27, 90). To assess self-reported interoceptive accuracy, we developed a question, which takes the heartbeat perception task by Schandry (88) into account: “How intense do you perceive your heartbeat in the moment?”. Based on previous studies (e.g., 99, 100), questions regarding mood comprise six positive emotions (cheerful, enthusiastic, awake, active, relaxed, calm) and five negative emotions (irritated, bored, nervous / stressed, worried, depressed), also assessed *via* a visual analogue scale ranging from 0 = “not at all” to 100 = “very much”.

##### 2.8.2.3. Mindfulness

Mindfulness is assessed *via* the 14-item short version of the Freiburg Mindfulness Inventory (FMI; 101). The FMI consists of a 4-point Likert scale ranging from 1 = “rarely” to 4 = “almost always” which are added to a sum score (range: 14–56). Higher scores indicate higher mindfulness. High internal consistency (Cronbach's Alpha = 0.84; 102) and sensitivity to change (101) were demonstrated.

##### 2.8.2.4. General anxiety

The 7-item Generalized Anxiety Disorder Questionnaire (GAD-7; 103) is used to screen for generalized anxiety disorders. Anxiety symptoms regarding the last two weeks are rated on a 4-point Likert scale ranging from 0 = “not at all” to 3 = “nearly every day”. A sum score (range: 0–21) is calculated. The GAD-7 has been proven as a reliable and valid measurement instrument of anxiety in the general population (Cronbach's Alpha = 0.89; 104). Furthermore, (103) reported a sensitivity of 0.89, a specificity of 0.82 and a good test-retest reliability (intraclass correlation = 0.83).

##### 2.8.2.5. Depression

The Patient Health Questionnaire (PHQ-8; 105) is an 8-item version of the reliable and valid PHQ-9 (106) without the ninth item requesting suicidal or self-injurious thoughts. The questionnaire assesses depressive symptoms within the last two weeks prior to measurement. Leaving out the ninth item might be more applicable in the context of an online- and smartphone-based study, as the handling with participants exhibiting suicidal or self-injurious thoughts without personal contact might be not adequate (105). A sum score (range: 0–24) is calculated from the ratings on a 4-point-Likert scale ranging from 0 = “not at all” to 3 = “nearly every day”. Based on a cutoff score of 10, the PHQ-8 showed a sensitivity of 0.86 (95% CI = 0.80–0.90) and a specificity of 0.86 (95% CI = 0.83–0.89; 107).

##### 2.8.2.6. Personality

The short version of the Big Five Inventory (108, 109) is used to measure the Big Five personality dimensions with two items per dimension. The 5-point Likert scale ranges from 1 = “fully disagree” to 5 = “fully agree”, which are calculated to a mean score. The questionnaire exhibited sufficient psychometric properties with an average retest reliability of 0.56.

##### 2.8.2.7. Emotion regulation

Emotion regulation is assessed *via* the German version (110) of the Emotion Regulation Questionnaire (111). The questionnaire comprises 10 items representing the two different emotion regulation strategies reappraisal (6 items) and suppression (4 items). Participants are instructed to rate the items on a scale ranging from 1 = “strongly disagree” to 7 = “strongly agree”. A higher mean for one of the two subscales reflects the preference for the respective strategy. Good internal consistencies were found for both subscales suppression (Cronbach's Alpha = 0.76) and for reappraisal (Cronbach's Alpha = 0.74; 110).

##### 2.8.2.8. Psychological well-being

The well-established 5-item WHO Well-Being Index (WHO-5; 112, 113) is utilized to assess subjective psychological well-being, in particular, the frequency of respective feelings over the last two weeks. Participants are instructed to response on a 5-point Likert scale ranging from 5 = “all of the time” to 0 = “at no time”. To calculate the total score, the sum score is calculated from raw scores (range: 0–25) and then multiplied with 4 (range: 0–100; 100 = best well-being). Based on several clinical studies, the WHO-5 demonstrated a sensitivity of 0.86 and a specificity of 0.81 as a screening tool for depression (113). A recent study (114) reported a Cronbach's Alpha of 0.75.

##### 2.8.2.9. Stress mindset

The Stress Mindset Measure (SMM; 76) is used to assess the individual general mindset if the effects of stress are enhancing or debilitating. The questionnaire consists of 8 items which the participants rate on a 5-point Likert Scale ranging from 0 = “strongly disagree” to 4 = “strongly agree”. Three optional preliminary questions address the current amount of stress (1 = “none”, 7 = “an extreme amount”), the primary source of stress in the individual's life and as how stressful this is perceived (1 = “not stressful”, 7 = “extremely stressful”). SMM scores are computed by means including the reverse scoring of four negative items. Higher scores indicate the mindset that stress is enhancing. The questionnaire demonstrated good internal consistency with Cronbach's Alpha = 0.86, which is similar for the German version (76, 115).

##### 2.8.2.10. Treatment expectancy

Treatment expectancy regarding the intervention is measured by the Credibility Expectancy Questionnaire (116), adapted for the chatbot-based intervention. Prior to randomisation, participants of the intervention as well as of the control group rate four items on a 9-point Likert scale and two items on a 10-point Likert scale. The scale reflects the two factors “credibility” and “expectancy”. Higher mean scores represent positive credibility and expectations. Cronbach's Alpha of 0.84 to 0.85 for the total scale indicated high internal consistency (117).

##### 2.8.2.11. Affinity for technology

The affinity for technology as an interaction style with technical systems, based on the established psychological construct need for cognition, was assessed *via* the Affinity for Technology (ATI) Scale (118). In this context, “technical systems” refer to apps, software applications or digital devices, respectively, the chatbot-based platform in the present study. Nine items are rated on a Likert scale ranging from 1 = “completely disagree” to 6 = “completely agree” and are calculated to an overall mean score. Based on multiple studies, the ATI Scale exhibited good psychometric properties regarding reliability (Cronbach's Alpha between .83 and .92), validity, dimensionality and distribution of ATI score values (118).

##### 2.8.2.12. Attitude towards artificial intelligence

The attitude towards artificial intelligence is assessed *via* the 5-item Attitude Towards Artificial Intelligence (ATAI) Scale (119). The according 11-point Likert scale ranges from 0 = “strongly disagree” to 10 = “strongly agree”. The ATAI Scale comprises the two factors “acceptance” and “fear”. A total mean score is calculated based on the means of the two subscales. In a study with a German sample (119), internal consistency for the subscale “acceptance” of artificial intelligence was at Cronbach's Alpha = 0.65 and for “fear” of artificial intelligence at Cronbach's Alpha = 0.66.

##### 2.8.2.13. Satisfaction with the intervention

To assess the global satisfaction with the intervention, a German version of the Client Satisfaction Questionnaire (CSQ-8; 120) adapted for the evaluation of internet-based interventions (121) was used. The CSQ-8 comprises eight items with diverse 4-point rating scales regarding the satisfaction such as 1 = “No, definitely not”, 4 = “Yes, definitely”, or 1 = “quite dissatisfied”, 4 = “very satisfied”. A sum score is computed with higher scores indicating higher satisfaction. Internal consistency of the CSQ-8 has been identified as high with Cronbach's Alpha between 0.88 and 0.92 (120, 122). In a study based on two randomised control trials investigating web-based interventions, results showed a good overall psychometric quality of the CSQ-8 (121).

##### 2.8.2.14. Mental health app usability questionnaire

The 18-item Mental Health App Usability Questionnaire (MAUQ; 75) is used to measure the usability of the chatbot as a mental health App, consisting of the three subscales „ease of use“ (5 items), „interface and satisfaction“ (7 items) and „usefulness“ (6 items). The rating scale ranges from 1 = “strongly agree” to 7 = “strongly disagree”. Mean scores for each subscale and a total mean score are calculated. The lower the mean score, the higher the usability. For the present study, a self-translated German translation of the MAUQ is used. The MAUQ exhibited an excellent internal consistency (Cronbach's Alpha = 0.91, 75).

##### 2.8.2.15. Negative effects

The Inventory for the Assessment of Negative Effects of Psychotherapy (INEP; 123) assesses potential negative effects of psychotherapy (e.g., intrapersonal change, relationships, or stigmatization). The scale comprises 21 items, including four items which are rated on a 7-point bipolar scale (−3 = “worse; + 3 = “better”), calculated to mean scores with lower values reflecting more negative effects. The other items are rated on a 4-point Likert scale ranging from 0 = “not at all” to 3 = “totally agree”. In the present study, an 18-item version adapted to possible negative effects of the chatbot-based intervention (e.g., excluding items regarding the therapist) is used to assess potential negative effects of the chatbot-based intervention. The original questionnaire exhibited a high internal consistency with a Cronbach's Alpha of 0.86 (123).

##### 2.8.2.16. Adherence

According to the suggested guidelines regarding adherence in randomised controlled trials investigating online interventions (124), adherence to the intervention is operationalized by the percentage of completed intervention units. All assessed variables, the according measurement instruments and measurement points are depicted in Table 2. Reasons for potential dropout reasons are assessed *via* the Dropout Reasons Questionnaire for Internet Interventions (125).

**Table 2:** Assessed variables, measurement instruments and measurement points.

Variables	Measurement instrument	Measurement point				
		T0	T1	T1–T2	T2	T3
Primary outcome						
Acute perceived stress	Perceived Stress Scale (PSS-10)	X				
Acute perceived stress	Perceived Stress Scale - short form (PSS-4); 2 items (EMA)		X	X	X	X
Secondary outcomes						
Demographic variables (e.g., age, gender, pre-experience with chatbots)	Demographic Questionnaire		X			
Self-reported interoceptive accuracy	Interoceptive Accuracy Scale		X		X	X
Self-reported interoceptive accuracy	Awareness Scale of the Body Perception Questionnaire		X		X	X
Self-reported interoceptive accuracy	Interoceptive item (EMA)			X		
Body Awareness	Body awareness items (EMA)			X		
Mood	Visual Analogue Mood Scale (EMA)			X		
Mindfulness	Freiburg Mindfulness Inventory – short form (FMI)		X		X	X
General anxiety	General Anxiety Disorder (GAD-7)		X		X	X
Depression	Patient Health Questionnaire (PHQ-8)		X		X	X
Personality	Big Five Inventory (BFI-10)		X			
Emotion regulation	Emotion Regulation Questionnaire (ERQ)		X		X	X
Psychological well-being	WHO-5 Well-Being Index		X		X	X
Stress mindset	Stress Mindset Measure (SMM)		X		X	X
Intervention credibility and expectancies	Credibility / Expectancy Questionnaire (CEQ)		X			
Affinity for Technology	Affinity for Technology Interaction (ATI)		X			
Attitudes towards Artificial Intelligence	Attitudes towards Artificial Intelligence (ATAI) Scale		X		X	X
Satisfaction with the intervention	Client Satisfaction Questionnaire (CSQ-8)				X	
Usability of the app	Mental Health App Usability Questionnaire (MAUQ)				X	
Negative effects of the intervention	Inventory for the assessment of Negative Effects of Psychotherapy (INEP)				X	X
Adherence	Percentage of completed intervention units				X	
Dropout reasons	Dropout Questionnaire				X	
Open feedback questions regarding the chatbot					X	

Notes. EMA, ecological momentary assessment.

### 2.9. Sample size estimation

The sample size was calculated by an a-priori power analysis for a repeated measurement ANOVA *via* G*Power (126), comparing two groups. Assuming a small effect size of *f* = 0.15 (consistent with *d* = 0.30), based on an *α*-level of 0.05, a power of 0.90, and a dropout rate of 40%, the sample size analysis resulted in *N* = 130 participants (*n* = 65 in the intervention group, *n* = 65 in the control group). As previous findings regarding chatbot-based interventions in the area of mental health are sparse, the assumed effect size of *d* = 0.3 was determined based on a systematic review (127), reporting effect sizes of *d* = 0.29 for depression and *d *= 0.15 for anxiety. A review and meta-analysis based on online mindfulness-based interventions stated small effect sizes for mindfulness (*g* = 0.32), depression (*g* = 0.29), anxiety (*g* = 0.22), and well-being (*g* = 0.23). It needs to be noted that results regarding adherence, respectively, attrition rates in online or chatbot-based interventions are also sparse or often not reported (124, 55). The dropout rate of 40% is estimated based on the systematic review of adherence to web-based interventions (128) and the adherence rates of guided interventions (39, 45, 46).

### 2.10. Data analysis

Multilevel modelling will be applied to analyse the longitudinal, nested data structure and change over time. The data analyses will be conducted according to the intention-to-treat principle. Procedures of imputation will be chosen based on patterns of missingness. The significance level for all analyses will be *p* ≤ 0.05. Exploratory mediation and moderator analyses including the primary and secondary outcomes and demographic variables will be conducted to examine how individual growth will be mediated or moderated by the according variables.

## 3. Discussion

The present study is designed to investigate the effects of a three-week chatbot-based intervention *via* smartphone, aiming to reduce stress and to improve various health-related parameters such as interoception in participants with medium to high stress levels. To the best of our knowledge, this is the first chatbot-based intervention addressing interoception, as well as in the context with the target variables stress and mindfulness. Strengths of the study are the design as a two-arm randomised controlled trial with a treatment as usual control group and outcome assessments pre-, post- and follow-up intervention as well as ecological momentary assessments, which is quite new in the assessment of interoception (90, 129, 130). Moreover, the highly standardized design is in line with the CONSORT guidelines (71, 73). In this context, the design of the present study and the usability of the chatbot was successfully tested in a previous feasibility study to establish a high quality, data security and usability of the intervention. Therefore, the user feedback had been implemented into the adapted version of the internally developed chatbot and will be analyzed to even potentially improve the intervention. Beyond that, the present study could shed light on the development of chatbots in the mental health area, in particular, for a stressed target group.

A possible limitation could be a limited attrition rate, as there is, for example, the issue of lacking long-term user engagement in e-Health (53, 131). One important factor in this context might be the feeling not to interact with a “real” human (132). At the same time, it needs to be highlighted that adherence rates of online or chatbot-based interventions are often not reported or were operationalised by diverse assessments (55, 124). To counteract a low adherence of the chatbot-based intervention, we implemented high guidance by the chatbot, short sessions, individual and flexible time points of the intervention units and the ecological momentary assessments, reminder SMS, and the opportunity to postpone single units. At the same time, the intervention is supposed to be used in everyday life, i.e., it should be provided in real time, in a natural setting, diverse contexts, comparably to ecological momentary interventions (133, 134). Additionally, the intervention contains personalised elements which are considered to be essential for chatbots in the area of health care, e.g., for user satisfaction and user engagement (135). Moreover, 53 concluded the significance of usability and interactivity in the context of mental health technology. Further limitations of the study might consist in the risks of measurement reactivity, especially in the context of digital ecological momentary assessments (136), or the systematic self-report bias in health data (137) due to self-report measures only. Moreover, as reported in the study by (138), differences to physiological assessments are possible, which are not part of the current study. To address the challenges of a potential gender bias and self-selection by participants with a high technical or online affinity, the chatbot was named gender-neutral, no avatar image of the chatbot is presented, and broad, nationwide recruitment strategies are realized (e.g., 65).

## Ethics and dissemination

The trial has been approved by the ethics committee of Ulm University (No. 401/20) and registered in the German Clinical Trials Register (DRKS00027560) on 06 January 2022. Participants in the study will receive written information on study conditions, data security, voluntary participation, the right to leave the study at any time, and the publication of anonymised results. Written informed consent will be obtained from all participants prior to their participation. In the present study, only self-report data but no psychophysiological data will be assessed. Data collection by the chatbot will happen on a secure on-premises server with limited access by a single team member. Online questionnaires *via* Unipark will be pseudonymised and linked to the conversations with a random token. All personal information as well as the tokens’ coding list will only be stored on the secure server and will be deleted after the study is completed. Only pseudonymised data is stored on a secured cloud storage with restricted access to the remaining authorised study personnel obliged to secrecy. According to German law, data will only be shared with parties outside the project team in anonymized form. Trial results will be submitted for publication in a peer-reviewed journal and presented at conferences.

## Author contributions

CS initiated the study. ELME was developed by the Department of Clinical and Health Psychology and the Institute of Distributed Systems at Ulm University (lead developers CS, DM and BE). CS, DM, BE, DS and OP designed and planned the study. DS, EB and OP supervised the study. CS is responsible for the recruitment and the conduction of the study. DM is responsible for the technical implementation of the chatbot. CS wrote the first draft of the manuscript. All authors contributed to the article and approved the submitted version.
